# Recurrent GANs Password Cracker For IoT Password Security Enhancement †

**DOI:** 10.3390/s20113106

**Published:** 2020-05-31

**Authors:** Sungyup Nam, Seungho Jeon, Hongkyo Kim, Jongsub Moon

**Affiliations:** Graduate School of Information Security, Korea University, Seoul 02841, Korea; synam@korea.ac.kr (S.N.); ohgnu90@korea.ac.kr (S.J.); oxqo@korea.ac.kr (H.K.)

**Keywords:** password cracking, GAN, IWGAN, RNN, PCFG, passGAN, hashcat, IoT

## Abstract

Text-based passwords are a fundamental and popular means of authentication. Password authentication can be simply implemented because it does not require any equipment, unlike biometric authentication, and it relies only on the users’ memory. This reliance on memory is a weakness of passwords, and people therefore usually use easy-to-remember passwords, such as “iloveyou1234”. However, these sample passwords are not difficult to crack. The default passwords of IoT also are text-based passwords and are easy to crack. This weakness enables free password cracking tools such as Hashcat and JtR to execute millions of cracking attempts per second. Finally, this weakness creates a security hole in networks by giving hackers access to an IoT device easily. Research has been conducted to better exploit weak passwords to improve password-cracking performance. The Markov model and probabilistic context-free-grammar (PCFG) are representative research results, and PassGAN, which uses generative adversarial networks (GANs), was recently introduced. These advanced password cracking techniques contribute to the development of better password strength checkers. We studied some methods of improving the performance of PassGAN, and developed two approaches for better password cracking: the first was changing the convolutional neural network (CNN)-based improved Wasserstein GAN (IWGAN) cost function to an RNN-based cost function; the second was employing the dual-discriminator GAN structure. In the password cracking performance experiments, our models showed 10–15% better performance than PassGAN. Through additional performance experiments with PCFG, we identified the cracking performance advantages of PassGAN and our models over PCFG. Finally, we prove that our models enhanced password strength estimation through a comparison with zxcvbn.

## 1. Introduction

As the computing power of IoT devices such as drones and smartwatches have improved, they have been utilized not only for entertainment purposes but also in various fields, such as military services and delivery services. Such enhanced computing ability allows the devices to operate on a modern operating system (OS) such as Linux that contains various applications, including a file transfer protocol (FTP), which is susceptible to massive network-based attacks such as a distributed denial of service (DDoS) attack [1]. Thus, proper security mechanisms for IoT devices are required to avoid the abuse of the devices. Among many security mechanisms, password-based authentication, especially text-based passwords, is a popular choice. The text-based password is a basic and fundamental authentication method, and it often plays a crucial role in system security. Passwords are both easy to understand and use and easy to implement [2]. Password authentication depends only on people’s ability to remember their passwords but does not need any additional equipment. This characteristic makes passwords the most popular authentication method, including for IoT devices, and passwords are often used as the first authentication method in multi-factor authentication. The fact that a password relies on a user’s ability to remember can also be a weakness. The dependence on memory induces people to use passwords in easy-to-remember word patterns [3,4,5]—for example, “iloveyou12”, and these are easily cracked by rule-governed dictionary-based password attacks. Using easy-to-remember passwords or known default passwords makes the device vulnerable to a security breach, as well as more sophisticated attacks involving IoT devices such as DDoS. To improve on the weakness of these passwords, password strength estimation studies have been conducted to recommend the usage of strong passwords, and organizations such as the National Institute of Standards and Technology (NIST) recommend the use of strong passwords at the policy level [6]. As a strong password usage policy, major sites such as gmail.com and microsoft.com enforce the use of a combination of uppercase and lowercase letters, digits, and special characters in passwords.

To crack high-complexity passwords, a password cracking method is required beyond a rule-based dictionary attack, and research into the development of such a technique is needed. The following is a detailed description of the cases in which password cracking technology needs to be developed. The first case is that people often forget their passwords; in particular, if a user sets a complicated password (which is unlike a pattern used in the past), the user will forget it more quickly and will need an effective password cracking method. Second, national agencies may need to crack passwords to obtain encrypted criminal evidence or intelligence information. Finally, valid password cracking methods are needed to secure passwords properly. Password cracking techniques can be used to estimate the strength of passwords practically. The zxcvbn method [7], which is used in DropBox, uses simple password crackers to estimate password strength. In this paper, we focus on improving password cracking performance rather than evaluating password strength.

Password cracking methods can be broadly divided into two types: the first type is the brute-force attack—i.e., an exhaustive attack—while the dictionary-based attack is the second type, which also has a hybrid attack variation [8]. The hybrid attack applies transformation or adds masking to the attack dictionary. Leaked passwords or an English dictionary are usually used as an attack dictionary. In some cases, a hybrid attack is distinguished from dictionary-based attacks, but in this study, we consider hybrid attacks to be dictionary-based attacks in a broad sense from the perspective of an expanded dictionary-based attack. The brute-force attack (or exhaustive attack) is a method of cracking a password by generating all the character combinations within a given password length range. The number of hash calculations per second executed in a brute-force attack has increased due to the development of GPU technology and the development of heterogeneous computing technology. In addition, if the time required for hash calculations such as MD5 or NTLM is short, the number of operations per second is high, meaning that the crack efficiency is good [9]. However, the recent use of slow hash algorithms such as bcrypt and md5crypt has resulted in poor cracking efficiency for the brute-force attack [9]. The dictionary-based cracking option involves the preparation of a word or combination of words that are likely to be used as a password, the calculation of the hash value of this item, and the comparison of the hash to the cracking target. Although cracking can be done at a constant rate regardless of password length, the disadvantage is that the password cracking search range is limited to the dictionary used for the attack. To overcome this, a method for defining and utilizing password transformation rules or adding masking rules to broaden the password search range is used. Hashcat [10] and John the Ripper (JtR) [11] are the most popular free tools for password cracking, and they support all of the password attack methods described above.

In this study, we focus on dictionary-based attacks. The dictionary-based password attack can be said to be a data-based attack, and the definition and use of dictionary transformation rules supported by Hashcat [10] and JtR [11] can be said to be the most basic data-driven expanded attack. This is because the password transformation rules are used by experts to analyze leaked passwords, identify frequently used transformation rules, and describe them with a specific format.

However, the methods used by security experts to analyze leaked passwords and create rules have limitations: the first is that the number of leaked passwords is too large for people to analyze. There are about 14 million plaintext passwords in RockYou [12,13], and there are about 60 million LinkedIn [14] plaintext passwords cracked at Hashes.org. Analyzing tens of millions of leaked passwords to create password transformation rules is time-consuming and tedious for people to do. Second, it is challenging to cover tens of millions of leaked password objects with thousands of rules. Because passwords reflect personal preferences and linguistic characteristics (Korean, Japanese, Chinese, etc.), it is challenging to represent all possibilities with specific rules, and if as many rules are generated as the number of leaked passwords, the efficiency of password cracking will vanish. Therefore, a study was conducted to automatically generate a password that would be likely to be used by people by analyzing a leaked password. The probability-based analysis of leaked passwords was conducted to generate new passwords, followed by template-based password generation studies using probabilistic context-free grammar (PCFG) [15,16]. With the development of machine learning, especially deep learning technology, studies on the application of deep learning technology to password cracking have recently been published. The most recently published study was PassGAN [17], and our team has introduced rPassGAN, which applied the recurrent neural network (RNN) to PassGAN. In this study, we present an additional RNN-based PassGAN model and confirm the superiority of the RNN-based deep learning model through a performance comparison between deep learning models. In addition, through a performance comparison analysis with PCFG, which showed excellent performance overall, we identified the complementary characteristics of PCFG and deep learning models.

The structure of this manuscript is as follows. In Section 2, we briefly explain the study of probability-based password cracking methods, including PCFG, as well as providing basic knowledge about deep learning and PassGAN. In Section 3, we first describe the model we presented in previous research, and then we investigate the disadvantages of the previous model. We explain how we tried to solve the problem with the previous model, and we propose a new model that does not suffer from the same problem. Section 4 describes the experimental environment and the process of deriving deep learning parameters. In Section 5, a performance comparison between deep learning models and a performance comparison that includes PCFG is presented; in this section, we further identify the causes of the performance gap between the PCFG and rPassGAN models in a performance comparison with PCFG. The final section presents the conclusions of this study.

## 2. Related Studies

In the following section, an assessment of password prediction methods will be introduced. A detailed analysis of the generative adversarial networks (GANs) will be provided in Section 3.

### 2.1. Markov and Context-Free Grammar Approaches

In using a probabilistic approach, a process using the Markov model was recommended. Narayanan and colleagues put forward a process that would create a pseudo password, working with the Markov model [18]. The central premise of this approach is that when people create a new password, they generally use simple passwords that they can remember easily; these passwords use a regular pattern of various alphanumeric sequences, which defines the probability of each sequence that is created. The research was subsequently extended by Ma et al. and Dürmuth et al. [4,5]. Ma attempted to find a better way of measuring password cracking performance and introduced the N-gram model for statistical language modeling to password modeling. Ma [5] showed that whole-string Markov models outperformed PCFG in their experiments. Dürmuth [4] also proposed an efficient password guessing program based on a Markov model, which was called OMEN. OMEN used an improved enumeration algorithm, which was called “enumPwd”, which allowed OMEN to produce the most likely passwords first. The application of the PCFG approach to the password prediction process, which was initially suggested by Weir and colleagues [15], was the most crucial aspect of these experiments. This PCFG-based analysis has continued to broaden and improve [19,20]. The current complex password grammatical structure is a combination of alphanumerical sequences, special characters, and keyboard-walks. This grammatical structure is used to analyze and calculate the distribution probability from leaked passwords. This information is then stored for later use. The PCFG generates a password using the grammatical structure with the highest probability using the information from the leaked passwords; this means that the PCFG improves its cracking capability over time. Methods using PCFG succeeded in cracking passwords at a higher rate when compared to attack methods that used a dictionary with Hashcat’s built-in rules as its source. In turn, the password cracking range has been effectively expanded by the use of this method. Houshmand and colleagues [20] focused on improving the cracking performance of keyboard walk structures and employed a smoothing technique. In their experiments, they showed better performance for cracking keyboard-walk patterned passwords than their previous approach.

### 2.2. Deep Learning Approaches

The RNN model of password prediction is a deep learning method that states that the characters that constitute the password are based on formerly used characters. This deep learning approach, which is comparable to the Markov method, performs remarkably well in the area of natural language processing and is often used in numerous functions, such as chat-bot applications, translation, and auto-completion. Melicher and colleagues [21] proposed a password prediction process that employs deep learning by applying an RNN [22]. This password prediction process creates a character unit password by using leaked passwords as training data. Furthermore, Hitaj and his colleagues developed the PassGAN [17] method, which also uses a deep learning-based password prediction process.

The relatively new and improved Wasserstein GAN (IWGAN) [23] was utilized in the creation of the PassGAN method. The GAN, which is used in PassGAN, is a deep learning model that has become the most popular generation model. Presented by Goodfellow and colleagues [24], the intent of the first GAN model was to create examples that they expected the population to include throughout the exercise. This was done to achieve a distribution that was indistinguishable from the high-dimension population. The GAN contains a unique structure compared to the current neural network model. The GAN applies two deep neural networks (DNNs) known as generative DNN (denoted as G) and the discriminative DNN (denoted as D): the role of G is to create perfect pseudo password samples that are indistinguishable from the authentic passwords, while the role of D is to differentiate between authentic passwords and the pseudo password samples created by G. This minimax problem, which was proven mathematically by Goodfellow, has a global optimum when the distribution of pseudo samples created by G is indistinguishable to the distribution of the factual data. The minimax problem can be expressed as follows:

(1) $$\underset{G}{min}\;\underset{D}{max}\;V\left(D,G\right)=\underset{x∼P_{data}\left(x\right)}{E}\left[logD\left(x\right)\right]+\underset{z∼P_{z}\left(z\right)}{E}\left[log\left(1-D\left(G\left(z\right)\right)\right)\right]$$

Multiple GAN models with improved performance have been suggested since Goodfellow first proposed his GAN model. The Wasserstein GAN (WGAN) [25] and the IWGAN [23] are two of the proposed GAN methods that offer the stability that allows the GAN model to find the global optimum. IWGAN was proposed by Gulrajani and colleagues [23], who introduced the concept of a gradient penalty instead of gradient clipping in WGAN and achieved stable training of the GAN model without divergence. The IWGAN showed that a CNN-based IWGAN model could be utilized for text generation. PassGAN utilizes RockYou leaked passwords [12,13] as training data and originated from the results of this experiment. In PassGAN, the role of D is to differentiate between the authentic passwords and the pseudo password samples created by G, while G is programed to create passwords that are comparable to the leaked passwords, which ultimately deceive D. In experiments, PassGAN displayed the ability to create passwords that Hashcat is unable to create [17].

Liu and his colleagues proposed the GENPass method, which uses both the generator and classifier as deep learning models for password generation [26]. The generator uses LSTM and PCFG. But the classifier uses CNN and heuristic constant. The discriminator judges whether a candidate will be accepted. In this paper, we will focus on the improvement of a deep learning model without using the multi-source approach.

## 3. Proposed Model

In our previous research [27], we approached the issue of improving password cracking performance from two standpoints. The first standpoint was to alter the discriminator and generator of the deep learning model, while the second was to add the structure change to our first approach.

### 3.1. Transformation of Neural Network Type

**Figure 1:**
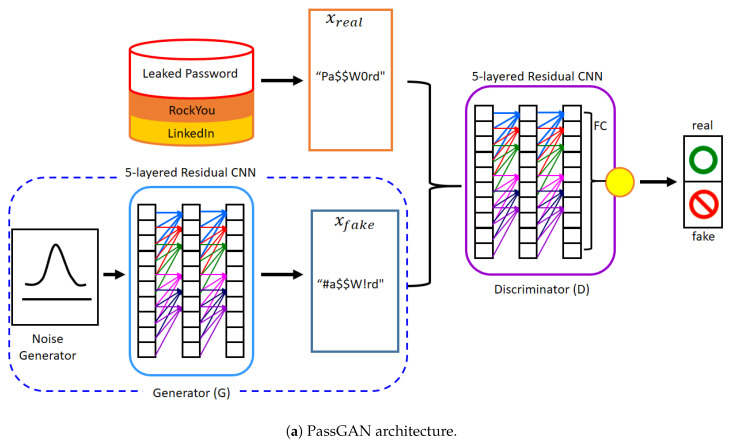

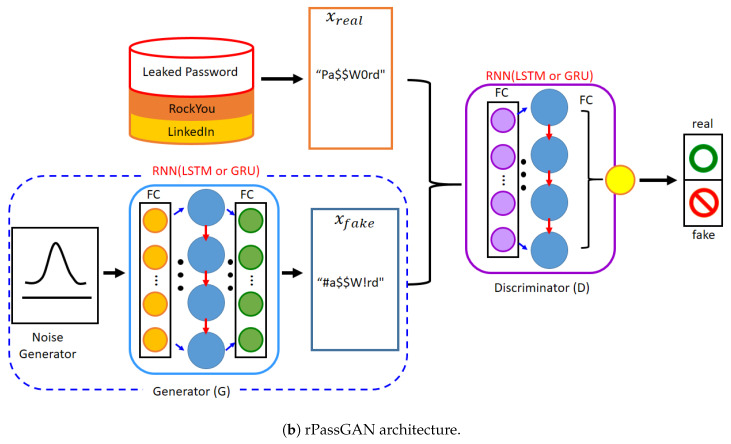
The model architecture of PassGAN & rPassGAN: PassGAN’s G and D consist of a five-layered residual convolutional neural network (CNN) with filter size 5. Otherwise, we replace the five-layered residual CNN with a one-layer RNN. In the rPassGAN, the red arrow stands for the recurrent flow. We mainly use gated recurrent unit (GRU) cells, because the training and sampling speed is better than long short-term memory (LSTM). FC stands for the fully-connected multilayer perceptron (MLP) in both Figure 1a,b. GAN stands for generative adversarial network.

Based on IWGAN, PassGAN was developed with the generator and the discriminator utilizing CNNs. The CNN, although not the ideal answer for text generation, can be used for this purpose. Passwords are created by characters that follow a certain pattern and obey specific rules. For example, consider the password “p@ssw0rd”: if we recognize the @ symbol and that letter s follows the letter p, then it is possible to infer that the letter s will be the next character. We arrive at this conjecture on account of people typically using characters in an established arrangement, with the preceding characters having a predictable result on the later characters. In this area of password cracking, the RNN [22] is a fitting deep-learning model that performs well in handling this sequential information, whereas long short-term memory (LSTM) [28] and gated recurrent unit (GRU) [29] are suitable RNN cell types. A few text generation studies have used the GAN model, which was based on LSTM or GRU [30,31,32]. Password cracking performance is likely to be improved when the model of D and G is shifted from a CNN model to an RNN model without modifying PassGAN’s design, as shown in Figure 1b. This new model is called rPassGAN.

### 3.2. Transformation of Architecture

**Figure 2:**
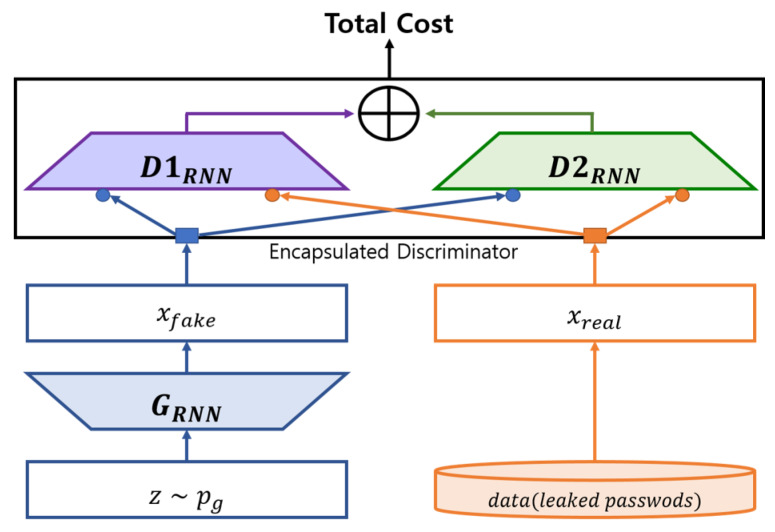
rPassD2CGAN block diagram.

We also proposed a change to the model of D and G from CNN to RNN, while simultaneously changing the structure of the GAN. When changing the GAN structure, we proposed the application of dual discriminators that utilize the IWGAN’s cost function. D1’s role is to identify the authentic passwords among the samples, whereas D2’s role is to identify the pseudo passwords among the samples. Both discriminators would execute the same role as in Nguyen and colleagues’ D2GAN method [33]. The objective of G in this dual-discriminator GAN model would be the deception of both discriminators, where the distribution of both the authentic and pseudo password samples created by G has to be indistinguishable. This creates a situation in which D1 and D2 are not able to differentiate between the authentic and pseudo passwords. Each discriminator’s cost is defined, in this first dual-discriminator model, with both discriminators being trained to minimize the cost. In contrast, the generator is trained to augment the total costs (Algorithm 1). This dual-discriminator combination PassGAN is known by its abbreviation rPassD2CGAN (Figure 2).
**Algorithm 1** rPassD2CGAN calculates each discriminator’s gradient penalty. We use the default values λ=10, $n_{critic}=10$, $n_{gen}=40$, α=0.0001, $\beta_{1}=0.5$ and $\beta_{2}=0.9$.**Require:** Gradient penalty coefficient λ, number of critic iterations per generator $n_{critic}$, number of generator iterations per discriminator $n_{gen}$, batch size *m*, and Adam hyper-parameters α, $\beta_{1}$, and $\beta_{2}$.
**Require:** Initial $D_{1}$ and $D_{2}$ critic parameters $w_{0}$ and $u_{0}$, and initial generator parameter $\theta_{0}$.
 **while**
θ has not converged **do**
  **for** 
$t=1,\ldots ,n_{critic}$ 
**do**
   **for** 
i=1,…,m 
**do**
    Sample real data $x∼P_{r}$, latent variable z∼p(z), and a random number ϵ∼U[0,1].
    $\tilde{x}\leftarrow G_{\theta }\left(z\right)$
    $\hat{x}\leftarrow \epsilon x+\left(1-\epsilon \right)\tilde{x}$
    $\bar{x}\leftarrow \epsilon \tilde{x}+\left(1-\epsilon \right)x$
    $L_{D_{1}}^{i}\leftarrow D_{w}\left(\tilde{x}\right)-D_{w}\left(x\right)+\lambda {\left({∥\nabla_{\hat{x}}D_{w}\left(\hat{x}\right)∥}_{2}-1\right)}^{2}$
    $L_{D_{2}}^{i}\leftarrow D_{u}\left(x\right)-D_{u}\left(\tilde{x}\right)+\lambda {\left({∥\nabla_{\bar{x}}D_{w}\left(\bar{x}\right)∥}_{2}-1\right)}^{2}$
    $L_{D2_{comb}}^{i}=L_{D_{1}}^{i}+L_{D_{2}}^{i}$
   **end for**
   $\left(w,u\right)\leftarrow \mathrm{Adam}\left(\nabla_{\left(w,u\right)}\frac{1}{m}\sum_{i=1}^{m}L_{D2_{comb}}^{\left(i\right)},w,u,\alpha ,\beta_{1},\beta_{2}\right)$
  **end for**
  **for** 
$t=1,\ldots ,n_{gen}$ 
**do**
   Sample a batch of latent variable ${\left\{z^{\left(i\right)}\right\}}_{i=1}^{m}∼p_{\left(z\right)}$
   $\theta \leftarrow \mathrm{Adam}(\nabla_{\theta }\frac{1}{m}\sum_{i=1}^{m}\left(-D_{w}\left(G_{\theta }\left(z\right)\right)+D_{u}\left(G_{\theta }\left(z\right)\right),\theta ,\alpha ,\beta_{1},\beta_{2}\right)$
  **end for**
 **end while**

### 3.3. Limitation of rPassD2CGAN

**Figure 3:**
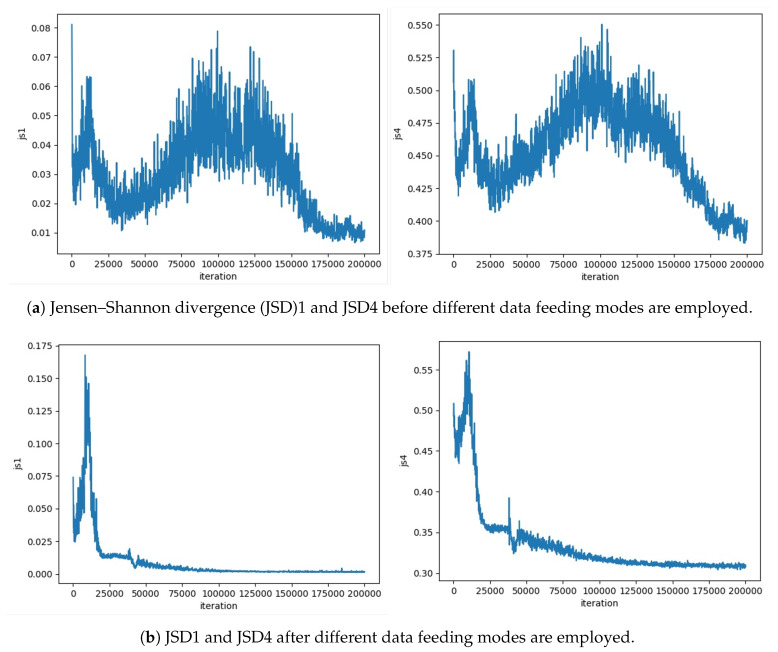
rPassD2CGAN showed some unstable training results. The use of different training data feeding modes stabilized rPassD2CGAN during training.

**Figure 4:**
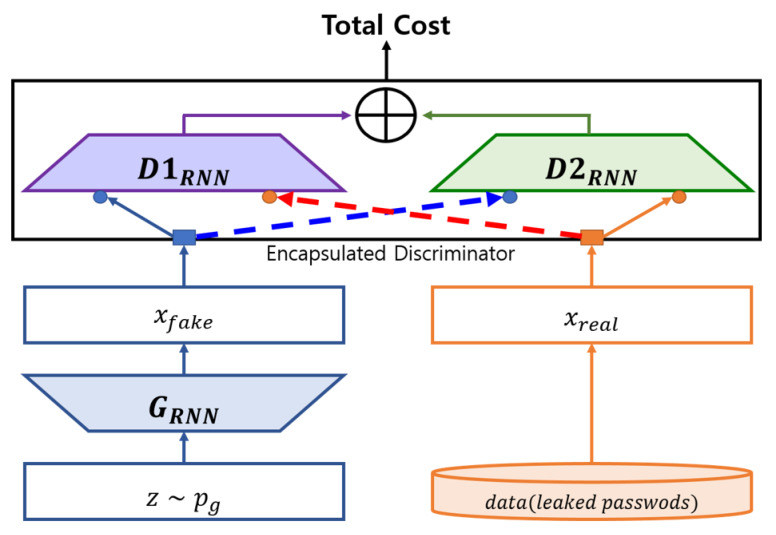
Different training data feeding modes.

rPassD2CGAN exhibited some unstable training results. The data used in the default training data feeding mode are identical in all discriminators. We provided D1 and D2 with the same fake and real passwords; however, when class 4 (number, special characters, alphabet lower and upper) long password data were provided, the rPassD2CGAN training status was unstable. Usually, the Jensen–Shannon divergence (JSD) reduces and then reaches an almost constant value. In the case of rPassD2CGAN, the JSD value did not converge but exhibited instability, as shown in Figure 3a. To solve this problem, we changed some hyper-parameters such as the batch size, G/D ratio, layer dimension, and learning-rate, but these measures were not effective. After many attempts, we decided to change the training data feeding modes. We tested two cases: one used the same real training data and different pseudo data for two discriminators, and the second used different real and pseudo training data feeding modes for two discriminators. These two training data feeding modes in Figure 4 enable rPassD2CGAN to carry out stable training. The password cracking results were similar but were not exactly the same.

### 3.4. New Dual-Discriminator Model

**Figure 5:**
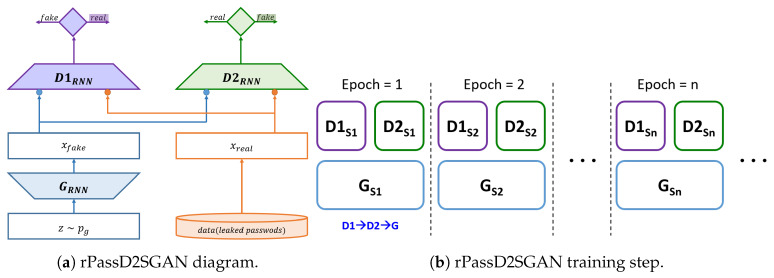
rPassD2SGAN block diagram and training step sequence.

Here, we introduce another dual-discriminator model that is slightly different from rPassD2CGAN. In rPassD2CGAN, two discriminators D1 and D2, were encapsulated; thus, the two discriminators operated as one discriminator, from the perspective of loss. In the second dual-discriminator model, D1 and D2 are not combined, and the hyper-parameters of D1 and D2 are trained sequentially. Algorithm  2 describes this operation. We use the same training parameter values of rPassD2CGAN for this model. We call this model rPassD2SGAN, and its block diagram is shown in Figure 5. The training sequence is D1, D2, and G, as shown in Figure 5b. rPassD2CGAN and rPassD2SGAN performed similarly in terms of their password cracking performance.
**Algorithm 2** rPassD2SGAN calculates each discriminator’s gradient penalty. We use default λ=10, $n_{critic}=10$, $n_{gen}=40$, α=0.0001, $\beta_{1}=0.5$, $\beta_{2}=0.9$.**Require:** The gradient penalty coefficient λ, the number of critic iteration per generator $n_{critic}$, the number of generator iteration per discriminator $n_{gen}$, the batch size *m*, Adam hyper-parameters α, $\beta_{1}$, $\beta_{2}$.
**Require:** initial $D_{1}$, $D_{2}$ critic parameters $w_{0}$ and $u_{0}$, initial generator parameter $\theta_{0}$
 **while** θ has not converged **do**
  **for** 
$t=1,\ldots ,n_{critic}$ 
**do**

   **for** 
i=1,…,m 
**do**

    Sample real data $x∼P_{r}$, latent variable z∼p(z), a random number ϵ∼U[0,1].    $\tilde{x}\leftarrow G_{\theta }\left(z\right)$    $\hat{x}\leftarrow \epsilon x+\left(1-\epsilon \right)\tilde{x}$

    $L_{D_{1}}^{i}\leftarrow D_{w}\left(\tilde{x}\right)-D_{w}\left(x\right)+\lambda {\left({∥\nabla_{\hat{x}}D_{w}\left(\hat{x}\right)∥}_{2}-1\right)}^{2}$
   **end for**

   $w\leftarrow \mathrm{Adam}(\nabla_{w}\frac{1}{m}\sum_{i=1}^{m}L_{D1}^{\left(i\right)},w,\alpha ,\beta_{1},\beta_{2})$
   **for** 
i=1,…,m 
**do**
    Sample real data $x∼P_{r}$, latent variable z∼p(z), a random number ϵ∼U[0,1].    $\tilde{x}\leftarrow G_{\theta }\left(z\right)$
    $\bar{x}\leftarrow \epsilon \tilde{x}+\left(1-\epsilon \right)x$
    
$L_{D_{2}}^{i}\leftarrow D_{u}\left(x\right)-D_{u}\left(\tilde{x}\right)+\lambda {\left({∥\nabla_{\bar{x}}D_{w}\left(\bar{x}\right)∥}_{2}-1\right)}^{2}$

   **end for**

   $u\leftarrow \mathrm{Adam}(\nabla_{u}\frac{1}{m}\sum_{i=1}^{m}L_{D2}^{i},u,\alpha ,\beta_{1},\beta_{2})$

  **end for**

  **for** 
$t=1,\ldots ,n_{gen}$ 
**do**

   Sample a batch of latent variable ${\left\{z^{\left(i\right)}\right\}}_{i=1}^{m}∼p_{\left(z\right)}$   $\theta \leftarrow \mathrm{Adam}(\nabla_{\theta }\frac{1}{m}\sum_{i=1}^{m}\left(-D_{w}\left(G_{\theta }\left(z\right)\right)+D_{u}\left(G_{\theta }\left(z\right)\right),\theta ,\alpha ,\beta_{1},\beta_{2}\right)$

  **end for**

 **end while**


In summary, we described two approaches in this section. The first is the neural network type transformation. PassGAN used CNN in the module (discriminator and generator); in contrast, our models used an RNN. This transformation changed the core architecture of PassGAN, and all hyper-parameters should be configured adequately for stable training. The second approach is the transformation of the architecture. D2GAN [33] used the integral probability metric (IPM)-based cost function, which is based on a cross entropy. GAN using the IPM-based cost function has a fundamental weakness; the probability of real data being real should decrease as the probability of fake data being real increases [34]. We applied D2GAN architecture using a non-IPM cost function (WGAN-GP) to rPassGAN. Furthermore, to solve the unstable training of the D2GAN architecture, a sequential training algorithm for dual discriminators was suggested.

## 4. Experiments

Experiments were devised to analyze if the prospective models have superior performance to that of PassGAN. Furthermore, the results were not only evaluated on their simple success rate at cracking the passwords, but also according to the extensibility of the cracked password. The exact experiment setup was as follows.

### 4.1. Training Configuration

#### 4.1.1. Training Parameters

An essential training framework equal to that of PassGAN (training values are detailed in Algorithm 1) was used in both models. Our experiments were conducted to obtain the suitable G/D training ratio for our models, with only a single G/D training ratio applied differently from that of PassGAN. The arrangement of the optimized G/D ratio experiment was as follows: the subjects of this experiment were three models that use RNN, excluding PassGAN. Utilizing data acquired from RockYou, the ratio of G/D training was increased from 10:10 to 100:10 with 20,000 (20k) training epochs. N-gram (N = 3, 4) JSD values of RockYou were contrasted with the dictionary generated by each RNN-based model to evaluate the results of the experiments.

**Figure 6:**
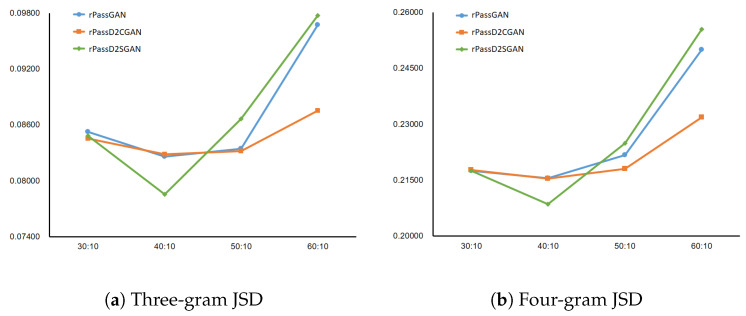
N-gram JSD (N = 3, 4) by G/D ratio (*X*-axis: G/D training ratio; *Y*-axis: JSD value). The data applied in training consisted of a RockYou password dictionary of length 1–8.

The lower the JSD value, the closer the value to the password distribution generated by people. In Figure 6, RNN-based models had the lowest JSD at 40:10 as G/D training iteration parameter. When we compared the cracking performances of rPassGAN, rPassD2CGAN, and rPassD2SGAN, we used the same hyper-parameters, such as the learning rate, G/D iteration ratio, and Adam optimizer parameters, to reduce other factors which may affect the performance.

#### 4.1.2. Training Data

Data in the form of plaintext passwords from RockYou were used during the training of the three deep-learning models. In this experiment, the RockYou dictionary was defined before the models were trained, and the Unicode characters were removed. Next, the length of the passwords found in the RockYou dictionary was analyzed, with nearly 14 million passwords with a length of 1 to 32 characters being used. These passwords were then separated into groups depending on their length, with training dictionaries created for each group using RockYou. The groups consisted of passwords with a length of 1–8 characters, 9–15 characters, and 16–32 characters, respectively. This was done to identify which model showed the best performance for each password length. In addition, when compared with PCFG, passwords of length 1–10 from RockYou and LinkedIn were used. This experiment condition replicated the experimental settings of Hitaj’s PassGAN [17].

### 4.2. Password Cracking

Passwords leaked from the LinkedIn website were used as the targets for our password cracking experiments. In total, 20% of the leaked LinkedIn passwords with a character length of 1–32 characters were used as the target in the password cracking performance test for the RockYou dictionary-trained models. Just as in the RockYou training data case, the Unicode passwords were removed from the password cracking targets. The best64 rule was used when testing the cracking performance with Hashcat. This was done for two reasons: first, conditions that are comparable to field conditions, where best64 is commonly applied to the password dictionary, were needed to test the performance of the password prediction methods; second, the billions of passwords utilizing the deep-learning model required a considerable amount of time to generate. Thus, even if a limited amount of passwords were generated, we could successfully crack many passwords that were identified with a smooth transformation by employing the best64 rule. As already mentioned in the above section, when comparing with PCFG, we did not employ the best64 rule to all models to create an experimental environment similar to that of PassGAN.

## 5. Evaluation

### 5.1. Dictionary Quality Perspective

**Figure 7:**
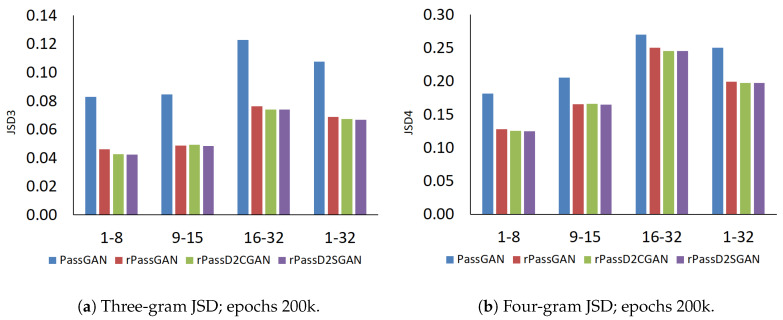
N-gram JSD of generated password dictionary (*X*-axis: password length; *Y*-axis: JSD value).

Two aspects were considered when we evaluated the quality of the password-prediction dictionary. The JSD was used as the evaluation criteria to measure the similarity of pseudo passwords generated by the model. First, the similarities between the dictionary generated by the model and the training dictionary created by people were considered. Second, the number of unique passwords predicted by the dictionary using the model was also considered. To avoid any degradation of the password cracking performance, the duplicated passwords generated by the model needed to be minimized as much as possible. The JSD value for each N-gram was compared with that of a human-generated password dictionary (the RockYou leaked password dictionary). As shown in Figure 7, the RNN-based models contained diminished JSD values compared to PassGAN for all N-grams. In terms of N-grams, the RNN-based models exhibited a higher resemblance to that human-generated password distribution than PassGAN.

The RNN-based models performed better than PassGAN when considering the rate of redundancy of the generated candidate passwords in Table 1. The space for generating the passwords widened exponentially when the length of the generated passwords increased. Therefore, the password group with the length of 16–32 characters saw its redundancy rate vanish. Even in a limited password space, the RNN-based models exhibited low password generation redundancy, causing the passwords to be cracked efficiently.

### 5.2. Cracking Performance Perspective

The large number of passwords successfully cracked and the contribution to the cracking of hashes that previously had not been cracked were the most appealing aspects of creating a password-guessing dictionary using models that were trained on a leaked-password dictionary. The improved password cracking performance and cracking area expansion capabilities of rPassGAN, rPassD2CGAN, and rPassD2SGAN compared with PassGAN will now be considered.

#### 5.2.1. Total Password Cracking

**Figure 8:**
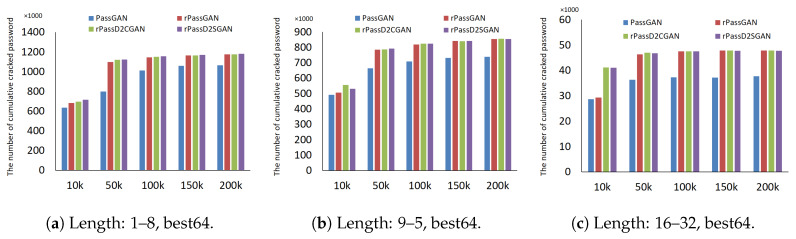
Total cumulative cracked passwords by training epoch.

First, the cracking performance for each training epoch will be presented in Figure 8. A modest contrast in password cracking performance after all models were trained for 100,000 (denoted as 100k) epochs was exhibited in the results of the RockYou password length groups. Acceptable password cracking performance for all password lengths and training epochs were exhibited in the RNN-based models.

**Figure 9:**
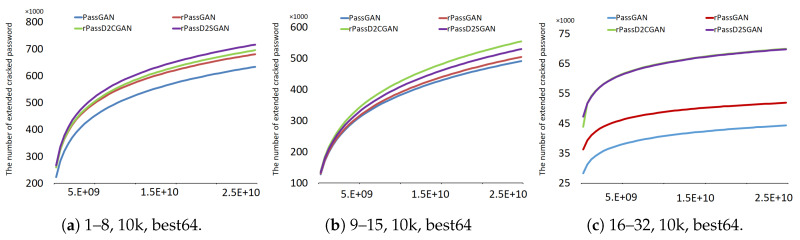
Total cumulative cracked passwords by the number of guessed passwords.

The connection between the number of password predictions and the number of increased password cracks is displayed in Figure 9. A deviation appears between the early training (10k epoch) model and the final (200k epoch) model when the number of increased password cracks is analyzed. At the beginning and even until the latter half of the experiment, both rPassD2CGAN and rPassD2SGAN display the highest number of increased crackings for the early training (10k epoch) model. When analyzing the 200k epochs, the only difference that can be confirmed is between PassGAN and the three RNN models due to the slim difference between the RNN models. A negligible difference can be seen in the results for the password-length groups. However, rPassD2CGAN and rPassD2SGAN performed better than rPassGAN in the case of the password length groups 9–15 and 16–32.

The password cracking performances between deep learning-based models were compared. Furthermore, we conducted password cracking performance measurement experiments that included PCFG, similar to Hitaj’s PassGAN study [17]. In these experiments, unlike in the previous experiments, we used RockYou and LinkedIn leaked passwords with the length 1–10 characters as training data and cracking targets. We created two sets of training data and cracking targets by using the Linux command “sort-R”. We split the randomly sorted data into training data (80%) and cracking targets (20%). Table 2 shows the password cracking performance for each model. “Tr” means training data, and “Cr” means cracking targets. Postfix digit means the index of the data set. For rk0–rk0 and rk0–lk0, deep learning models, including PassGAN, achieved similar or better password cracking performance than PCFG; except for these two cases, PCFG achieved the best performance.

We decided to measure JSD to estimate the difference in the probabilistic distribution between the training data and the cracking targets. Table 3 shows the result of the measurement. All cases except rk0-rk0 and rk0-lk0 showed a very low JSD value; in particular, JSD1 was almost zero. For rk0-rk0 and rk0-lk0, the JSD values were relatively high; the higher JSD values mean that the probabilistic distribution of the training data is quite different from that of the cracking target. This means that the deep learning models, including PassGAN, have the ability to crack passwords with new or strange patterns that did not exist in the training data.

#### 5.2.2. Cracking Extensibility

Below, the results of the password cracking space extension of each model’s password-predicting dictionary experiment are shown. Two perspectives were utilized when analyzing the experimental results of the password cracking space extensibility: first, the extension of the password dictionary generated by each model for the cracking space of the authentic passwords used in the training of each model was analyzed; second, the proportion of passwords that could be cracked by the model, and the number of passwords that the model did not crack for all proposed models, was compared.

Table 4 shows the disparity in the number of cracks generated by the password dictionary for each model. Regions in which all proposed models had exclusive password cracking success where the other models had none are also detailed. These data are significant, on account of the expansion of the password cracking space which the use of these dictionaries would create. rPassGAN was the model with the largest number of unique cracks, followed by rPassD2CGAN, rPassD2SGAN among our models. rPassD2SGAN had the largest number of unique cracks when compared with PassGAN. The RNN models exhibited a much smaller difference to each other than in comparison to PassGAN.

### 5.3. Password Strength Estimation

The models we selected for the password strength estimation experiment are zxcvbn [7], PCFG, and rPassGAN (with the minimum cracking performance model of the rPassGAN series). To estimate the entropy of passwords, the zxcvbn framework was basically used, because zxcvbn is practically used for password strength estimation for DropBox. Moreover, it has the function that external password candidates can be supplied to zxcvbn. With this function, the effectiveness of password candidates of both PCFG and rPassGAN could be analyzed as regards the estimation of the strength of passwords; if they were effective, the entropy of the password strength would be lower than that of zxcvbn’s basic mode [7]. For this experiment, we ran the password cracking experiment with the same configuration and training data. However, we decided to use a small amount of cracking target passwords for the estimation experiment in the case of the rk0 dataset. Since estimating the strength of 2 million passwords is time-consuming, we randomly made three 10k password sets from the rk0 dataset. In addition, we created some class 4 (uppercase letters, lowercase letters, digits, special symbols such as “!” and “@”) training and estimating datasets from RockYou, LinkedIn, iMesh, ZooSK, and Myspace leaked passwords. These cracked plaintext passwords can be downloaded from Hashes.org. In zxcvbn [7], neither KeePass nor NIST formalize the type of entropy they model; zxcvbn [7] assumes that *n* bits of strength means that guessing the password is equivalent to guessing a value of a random variable X according to

(2) $$n=H(X)=-\sum_{i}p\left(x_{i}\right)log_{2}p\left(x_{i}\right)$$

Assuming the guesser knows the distribution over *X* and attempts guesses $x_{i}$ in a decreasing order of probability $p(x_{i})$, a lower bound on the expected number of guesses E[G(X)] can be shown as

(3) $$E[G(X)]\geq 2^{H(X)-2}+1$$

provided that H(X)≥2. This conservative lower bound to convert bits into guesses in zxcvbn [7] was used. Table 5 shows the percentage of cases in which each model performs best, corresponding to the same passwords. A lower entropy and lower guessing number of a password mean that the strength of this password is not high; thus, if a model showed lower entropy than others, this model would represent a robust password strength estimator.

When the password candidates of rPassGAN and PCFG were supplied to zxcvbn, a better password strength estimating performance was observed. This means that rPassGAN and PCFG’s password candidates are helpful for stricter password strength estimation. Further, the entropy of rPassGAN was slightly lower than that of PCFG and zxcvbn with the rk0 dataset, where JSD is higher than the others (see Table 3). In the case of class 4 (password length 14–16), rPassGAN showed slightly lower entropy than PCFG except for passwords with a length of 16. In the case of cls4(16), rPassGAN showed weakness at estimating keyboard-walk passwords; in contrast, PCFG showed the advantages of estimating keyboard-walk passwords. In total, 20% passwords of PCFG’s best cases in cls4(16) were keyboard-walk passwords. rPassGAN should be trained with a collection of keyboard-walk passwords to make up for this weakness and generate the keyboard-walk password candidates, which would help zxcvbn in strictly estimating passwords. Table 6 shows some examples of password strength estimation results.

## 6. Conclusions

The three models introduced in this study performed better at cracking passwords than PassGAN—the first GAN-based password predicting model. We applied the RNN as the primary DNN model to our models. Although PassGAN performed better when changing from CNN to RNN, we achieved substantially improved performance with the adoption of a dual discriminator structure. From this experiment, we derived the G/D training ratio for the enhanced training in the RNN-based IWGAN. Through the use of password cracking performance tests, it has been shown that RNN-based models performed better than PassGAN. rPassGAN and PCFG both displayed improved password estimation results in the password strength estimation experiment. An extensive amount of time was needed to generate the password-guessing dictionary and train the exiting password dictionary using the deep-learning model; nonetheless, by performing these procedures, the time invested resulted in a secure password-predicting dictionary.

The selection of a password generation model for the password cracking extension of PCFG could be influenced by the results of this experiment. We advise the use of a single RNN-based model for password cracking when enough time is available, but the desired computing capability is not. The RNN-model chosen in this situation is not essential; however, if time is limited but adequate computer capability is available, the use of rPassD2CGAN or rPassD2SGAN is advised since both achieved sufficient password cracking performance with a small epoch, such as 10k. When a maximum number of cracked passwords is needed, all models should be used since each has its own set of cracked passwords. Although PCFG achieved a better cracking performance than the deep learning models, our results revealed that the deep learning models had advantages for certain password cracking cases; specifically, when the probabilistic distribution difference between the training and the cracking targets was significant, rPassGAN showed better cracking performance than PCFG. This means that the generality of the deep learning models was better than that of PCFG. Because PCFG as a model is suitable for cracking passwords that have patterns similar to those in training data, the first password-cracking step using PCFG is to crack passwords with similar patterns as the training data. In the second step, the deep learning model is used to implement password cracking with different learning data and patterns, which effectively maximizes the overall password cracking space. The maximized password candidates from both the RNN-based model and PCFG are helpful in setting default passwords for IoT devices. Furthermore, modern deep learning libraries, such as Google’s tensorflow [35], support the loading of the trained model with Javascript and for mobile devices. Thus, we can easily apply the deep learning password cracking methodology to web browsers and IoT devices.

## Figures and Tables

**Table 1:** Redundancy rate of password guessing candidate dictionary.

Models	1–8	9–15	16–32	1–32
PassGAN	7.52%	0.55%	0.10%	4.08%
rPassGAN	4.42%	0.15%	0.10%	2.02%
rPassD2CGAN	4.34%	0.15%	0.09%	2.05%
rPassD2SGAN	4.32%	0.15%	0.09%	2.05%

**Table 2:** Password cracking performance results.

Tr/Cr	PassGAN	rPassGAN	rPassD2CGAN	rPassD2SGAN	PCFG
rk0-rk0	251,592	287,745	289,753	292,626	141,278
lk0-lk0	666,571	735,264	764,762	764,082	2,725,501
rk0-lk0	677,542	765,324	772,956	777,586	2,186,212
lk0-rk0	346,303	391,565	399,256	402,696	417,802
rk1-rk1	396,851	440,020	449,401	448,772	845,539
lk1-lk1	707,567	732,795	753,700	763,494	2,724,828
rk1-lk1	738,499	803,508	824,561	823,639	2,505,716
lk1-rk1	299,449	314,142	321,967	324,575	961,771

Password guessing number: 250 million, with no Hashcat rule such as best64 employed, Tr means training data index; Cr means cracking data index. PCFG: probabilistic context-free grammar.

**Table 3:** JSD between training data and cracking target data in Table 2.

Tr/Cr	JSD1	JSD2	JSD3	JSD4
rk0-rk0	0.279475	0.347773	0.433052	0.536094
lk0-lk0	0.000000	0.000031	0.002026	0.030252
rk0-lk0	0.010636	0.024597	0.046771	0.096937
lk0-rk0	0.198082	0.250680	0.318582	0.415374
rk1-rk1	0.000001	0.000116	0.004643	0.041829
lk1-lk1	0.000000	0.000030	0.002032	0.030271
rk1-lk1	0.002210	0.013319	0.032067	0.079866
lk1-rk1	0.002188	0.013375	0.034495	0.095603

**Table 4:** Difference between each deep learning model’s cracking result set.

Models	PassGAN	rPassGAN	rPassD2CGAN	rPassD2SGAN
PassGAN	0	107,103	107,406	113,048
rPassGAN	235,937	0	143,182	149,495
rPassD2CGAN	235,127	142,069	0	148,390
rPassD2SGAN	241,456	149,069	149,077	0

The number of each element in the table stands for the size of the difference of the element between the two models. A better model has more unique cracked passwords; thus, for a better model, the size of difference will be larger than that of others. The elements are calculated by the following rule: *m*_1_ = PassGAN, *m*_2_ = rPassGAN, *m*_3_ = rPassD2CGAN, *m*_4_ = rPassD2SGAN $S_{m_{i}}$ = {x | x is cracked passwords of model *m_i_*} |*D*(*i*, *j*)| = |$S_{m_{i}}$ − $S_{m_{j}}$|, (i is row index, j is column index.) For example, |*D*(2, 1)| = |*S_rPassGAN_* − *S_PassGAN_*|.

**Table 5:** Percentage of the lowest entropy cases for each model.

Dataset	rPassGAN	PCFG	zxcvbn	None
rk0-0	15.6% (17.91)	2.3% (18.13)	0.5% (18.19)	81.6%
rk0-1	8.4% (31.84)	12.1% (31.79)	0.0% (32.37)	79.5%
rk0-2	12.3% (32.59)	12.3% (32.74)	0.0% (33.57)	75.4%
cls4(14)	4.7% (38.43)	5.7% (38.48)	0.1% (39.03)	89.5%
cls4(15)	8.4% (38.68)	4.9% (39.40)	0.1% (39.87)	86.6%
cls4(16)	1.4% (45.18)	4.6% (44.61)	0.8% (45.32)	93.2%

The stronger the password estimator is, the lower the entropy and guessing number will be. The values in parentheses are the average of all target passwords’ entropy for each model. The “None” column described the percentage of cases when the three models resulted in the same entropy value; this means that no models contributed to enhancing password estimation. The Prefix cls4 datasets were created from RockYou, LinkedIn, iMesh, ZooSK, and Myspace from Hashes.org. And, the number in the parentheses indicates the length of estimating passwords.

**Table 6:** The example results of password strength estimation, where rPassGAN showed the lowest entropy.

Passwords	rPassGAN	PCFG	zxcvbn
Charlotte.2010	18.83$\left(1.17\times 10^{5}\right)$	21.852 $\left(9.46\times 10^{5}\right)$	23.916 $\left(3.96\times 10^{6}\right)$
C0mm3m0r47!0ns	18.83 $\left(1.17\times 10^{5}\right)$	41.11 $\left(2.97\times 10^{11}\right)$	40.11 $\left(2.97\times 10^{11}\right)$
MyLinkedIn101!	25.333 $\left(1.06\times 10^{7}\right)$	28.605 $\left(1.02\times 10^{8}\right)$	35.598 $\left(1.30\times 10^{10}\right)$
Linkedin2011@@	17.897 $\left(6.10\times 10^{4}\right)$	19.167 $\left(1.47\times 10^{5}\right)$	27.644 $\left(5.24\times 10^{7}\right)$
Profe$$1ona11$m	18.527 $\left(9.44\times 10^{4}\right)$	29.824 $\left(2.38\times 10^{8}\right)$	29.824 $\left(2.38\times 10^{8}\right)$
Concep+ua1!z!ng	21.27 $\left(6.32\times 10^{5}\right)$	41.066 $\left(5.75\times 10^{11}\right)$	47.09 $\left(5.75\times 10^{11}\right)$
C0ns4nguini+i3s	14.997 $\left(8.18\times 10^{3}\right)$	47.09 $\left(3.74\times 10^{13}\right)$	47.09 $\left(3.74\times 10^{13}\right)$
September27,1987	22.682 $\left(1.68\times 10^{6}\right)$	40.457 $\left(3.77\times 10^{11}\right)$	40.457 $\left(3.77\times 10^{11}\right)$
JLN@linkedin2011	28.022 $\left(6.81\times 10^{7}\right)$	51.308 $\left(6.97\times 10^{14}\right)$	51.308 $\left(6.97\times 10^{14}\right)$
@WSX$RFV1qaz3edc	46.003 $\left(1.76\times 10^{13}\right)$	28.724 $\left(1.11\times 10^{8}\right)$	46.003 $\left(1.76\times 10^{13}\right)$
!QAZ1qaz@WSX2wsx	31.184 $\left(6.10\times 10^{8}\right)$	30.032 $\left(2.74\times 10^{8}\right)$	46.003 $\left(1.76\times 10^{13}\right)$
#$ERDFCV34erdfcv	55.142 $\left(9.94\times 10^{15}\right)$	24.188 $\left(4.78\times 10^{6}\right)$	55.142 $\left(9.94\times 10^{15}\right)$
1qaz)OKM2wsx(IJN	46.003 $\left(1.76\times 10^{13}\right)$	30.741 $\left(4.49\times 10^{8}\right)$	46.003 $\left(1.76\times 10^{13}\right)$
!QAZ#EDC%TGB7ujm	48.253 $\left(8.81\times 10^{13}\right)$	28.843 $\left(1.20\times 10^{8}\right)$	48.325 $\left(8.81\times 10^{13}\right)$

The number in parentheses is the guessing number from Equation (3). In keyboard-walk passwords, rPassGAN showed the weakness.

## Abbreviations

The following abbreviations are used in this manuscript:

GAN	Generative Adversarial Network
PCFG	Probabilistic Context-Free Grammar
RNN	Recurrent Neural Network
CNN	Convolutional Neural Network
JtR	John the Ripper
JSD	Jensen–Shannon Divergence
